# Autoinflammatory bone disorders with special focus on chronic recurrent multifocal osteomyelitis (CRMO)

**DOI:** 10.1186/1546-0096-11-47

**Published:** 2013-12-23

**Authors:** Christian M Hedrich, Sigrun R Hofmann, Jessica Pablik, Henner Morbach, Hermann J Girschick

**Affiliations:** 1 Division of Pediatric Rheumatology and Immunology, Children’s Hospital Dresden, University Medical Center Carl Gustav Carus, TU Dresden, Dresden, Germany; 2 Division of Pathology, University Medical Center Carl Gustav Carus, TU Dresden, Dresden, Germany; 3 Department of Pediatrics, University of Würzburg, Würzburg, Germany; 4 Children’s Hospital, Vivantes Klinikum-Friedrichshain, Berlin, Germany

**Keywords:** PAPA, DIRA, Majeed, CNO, CRMO, Bone, Inflammation, IL-10, TNF-α, TLR4, Treatment

## Abstract

Sterile bone inflammation is the hallmark of autoinflammatory bone disorders, including chronic nonbacterial osteomyelitis (CNO) with its most severe form chronic recurrent multifocal osteomyelitis (CRMO). Autoinflammatory osteopathies are the result of a dysregulated innate immune system, resulting in immune cell infiltration of the bone and subsequent osteoclast differentiation and activation. Interestingly, autoinflammatory bone disorders are associated with inflammation of the skin and/or the intestine. In several monogenic autoinflammatory bone disorders mutations in disease-causing genes have been reported. However, regardless of recent developments, the molecular pathogenesis of CNO/CRMO remains unclear.

Here, we discuss the clinical presentation and molecular pathophysiology of human autoinflammatory osteopathies and animal models with special focus on CNO/CRMO. Treatment options in monogenic autoinflammatory bone disorders and CRMO will be illustrated.

## Background

Autoinflammatory disorders are characterized by seemingly unprovoked systemic inflammation in the absence of auto-reactive T cells or high-titer auto-antibodies [1]. Sterile bone inflammation is the hallmark of autoinflammatory bone disorders, including pyogenic arthritis, pyoderma gangrenosum and acne (PAPA) syndrome, the deficiency of IL-1 receptor antagonist (DIRA), familial chronic multifocal osteomyelitis which is also referred to as Majeed syndrome, sporadic chronic recurrent multifocal osteomyelitis (CRMO), and synovitis, acne, pustulosis, hyperostosis and osteitis (SAPHO) syndrome [2,3].

Autoinflammatory bone disorders are the result of a disturbed regulation of the innate immune system, resulting in immune cell infiltration of the bone and subsequent osteoclast differentiation and activation, osteolysis and bone remodeling. Though bone biopsies usually remain sterile, lesions mimic infectious osteomyelitis in histology and on radiographs [2-5]. Interestingly, autoinflammatory bone disorders are associated with inflammation of the skin (palmoplantar pustulosis, acne, psoriasis, Sweet syndrome) and/or the intestine (Crohn’s disease, ulcerative colitis, coeliac disease) [2-5]. In several so-called monogenic autoinflammatory bone disorders mutations in disease-causing genes have been reported [1,6-8]. Regardless of recent developments, the molecular pathogenesis of sporadic CRMO, however, remains to be determined.

In the following, we discuss the clinical presentation and molecular pathophysiology of human autoinflammatory osteopathies and animal models with special focus on CNO/CRMO. Currently available treatment options in monogenic autoinflammatory bone disorders and CRMO will be illustrated.

## Review

### Monogenic autoinflammatory bone disorders

#### Pyogenic arthritis, pyoderma gangrenosum and acne (PAPA)

PAPA patients present during childhood with recurrent episodes of erosive arthritis, that in later disease stages may require joint replacement therapy. As patients progress to puberty, cutaneous involvement with neutrophilic skin lesions ranging from cystic acne to pyoderma gangrenosum may predominate [9]. Regardless of the clinical appearance, massively elevated cell numbers in the synovial fluid and the appearance of skin lesions suggesting an infectious process, cultures usually remain sterile. In a subset of patients, pathergy, irritable bowel syndrome, aphthous stomatitis and pancytopenia may occur [1-3].

**Figure 1:**
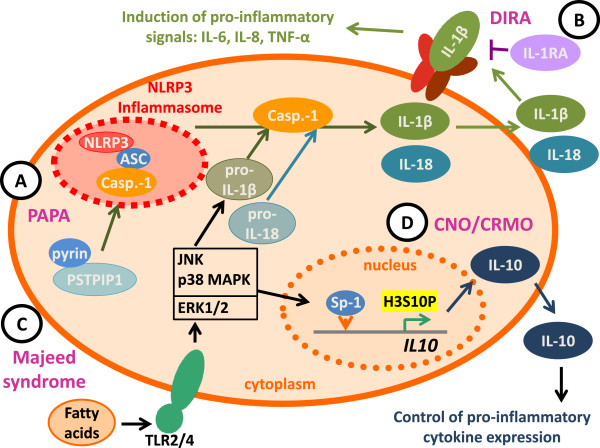
**The imbalance between pro- and anti-inflammatory cytokines is a hallmark of auto-inflammatory bone disorders. A)** The prolonged interaction between pyrin and PSTPIP1 in PAPA syndrome results in impaired inhibition of the NRLP3 inflammasome, resulting in enhanced IL-1β and IL-18 release after cleavage from pro-IL-1β/pro-IL-18 by activated caspase-1 (Casp.-1). **B)** In DIRA, a lack of functional IL-1 receptor antagonist (IL-1RA) results in impaired peripheral control of IL-1 signaling. **C)** In Majeed syndrome, Lipin2 deficiency may result in increased levels of fatty acids that may be recognized by TLR-2 and -4, resulting in Jun kinase (JNK) activation and subsequent pro-inflammatory signaling. **D)** In CRMO monocytes, impaired ERK1/2 activation results in reduced Sp-1 recruitment and decreased histone H3 phosphorylation (H3S10P) of the IL10 promoter. This molecular defect results in a failure to express IL-10 and an imbalance between pro- and anti-inflammatory cytokines.

PAPA segregates with mutations in the threonine phosphatase-interacting protein (PSTPIP)1 that is also referred to as CD2 binding protein (CD2BP)1 on chromosome 15 and follows an autosomal-dominant inheritance [8]. Interactions of the PSTPIP1 protein with the protein tyrosine phosphatase PEST results in phosphorylation of PSTPIP1 that in turn prevents its binding to pyrin, a negative regulator of the NLRP3 inflammasome. Mutations in PSTPIP1 result in impaired phosphorylation and subsequently prolonged interactions with pyrin, resulting in enhanced activation of the NLRP3 inflammasome [10-12] (Figure 1A). Alternatively it has been suggested that mutated PSTPIP1 may allow pyrin to oligomerize with adaptor proteins and form a pyrin inflammasome [11]. In both models, mutated PSTPIP1 mediates increased inflammasome activation and IL-1 (and IL-18) release, resulting in down-stream pro-inflammatory signaling through IL-8, IL-6, and TNF-α.

Diagnosis can be made based on the clinical picture and family history. Genomic testing for mutations in the PSTPIP1 gene is available in order to prove the clinical diagnosis. Treatment with corticosteroids, TNF-α inhibitors, and IL-1 blocking strategies bring clinical benefit. However, disease flares even occur in patients on high doses of IL-1 blockers, suggesting that IL-18, which is also cleaved by activated caspase-1, can sufficiently drive systemic inflammation in the absence of IL-1 [1,13]. Alternatively, an even more complex pathomechanistic role of PSTPIP1 in the molecular pathogenesis of PAPA may be discussed [11]. Waite et al. suggested that pyrin may be recruited into aggregations of apoptosis-associated speck-like protein containing a CARD (ASC), another pyrin-binding protein, which are then referred to as ASC specks. In the authors’ pathophysiological model ASC specks mediate particularly increased inflammasome activation and pro-inflammatory cytokine release [11].

#### Deficiency of IL-1 receptor antagonist (DIRA)

DIRA manifests in the first days of life with a pustular skin rash, joint swelling, painful resorptive bone lesions, and periostitis. Bone lesions usually affect distal ribs and long bones. In contrast to individuals suffering from cryopyrinopathies, DIRA patients usually do not present with fever. A subset of patients develop additional symptoms of central nervous system (CNS) vasculitis and interstitial lung disease [14].

DIRA follows an autosomal-recessive inheritance and is caused by mutations in the IL1RN gene on chromosome 2, encoding the IL-1 receptor antagonist (IL-1RA). The IL-1RA is a post-translational regulator of the pro-inflammatory cytokines IL-1α and IL-1β. IL-1RA competes with IL-1α and IL-1β for binding to the type I IL-1 receptor. Other than the IL-1 cytokines, IL-1RA prevents the simultaneous engagement of the IL-1 receptor accessory protein, thus blocking the formation of the active IL-1 receptor-signaling complex. Thus, IL1RN mutations in DIRA result in uninhibited IL-1αand IL-1β signaling (Figure 1B). To our knowledge three homozygous IL1RN mutations resulting in a stop codon have been reported in addition to 175 kb deletion including the IL1RN locus and five adjacent genes on chromosome 2q [14,15].

Diagnosis can be made based on the clinical picture with early onset dermatitis and osteitis in the absence of fevers. Genomic testing for mutations in the IL1RN gene is available. Patients rapidly respond to treatment with the recombinant IL-1RA anakinra. However, response may be incomplete in patients with the 175 kb genomic deletion, suggesting a contribution of the adjacent genes to the pathophysiology of DIRA [1,14,15].

#### Majeed syndrome

Familial inherited cases of chronic osteomyelitis, also referred to as Majeed syndrome, have been described four consanguineous families. Affected family members present with early-onset osteomyelitis within the first two years of life, a skin rash resembling Sweet syndrome, and a microcytic dyserythropoietic anemia. Usually, Majeed syndrome is more severe when compared with sporadic CRMO and may be accompanied by recurrent fevers [16,17].

Majeed syndrome follows an autosomal-recessive inheritance and has been linked with mutations in the LPIN2 gene, encoding for the Lipin2 protein [7]. Lipin2 is expressed in the liver, the kidneys, the gastrointestinal system, lymphoid tissues and the bone marrow. Interestingly, carriers of heterozygous LPIN2 mutations on one allele develop psoriatic skin lesions underscoring the potential pathophysiological linkage between skin involvement and bone inflammation [5,7,16-18]. Lipin2 is a phosphatidate phosphatase that plays a role in lipid metabolism. The exact function of Lipin2, however, remains to be established. Interestingly, Lipin2 shares sequence homology with Lipin1 that has been documented to carry polymorphisms and mutations in lipodystrophy in humans and mice. However, to date the involvement of Lipin2 in fatty acid metabolism cannot completely explain the inflammatory effects of mutations in Majeed syndrome [19-21]. Even though the phosphatase activity of Lipin2 is completely abolished in Majeed patients carrying the LPIN2 p.S73L mutation, its role in the control of inflammatory responses is less clear. Based on findings in the field of obesity-induced chronic inflammation, it has been suggested that fatty acids may induce a pro-inflammatory phenotype by toll-like receptor (TLR)2 and 4 stimulation that result in the activation of Mitogen Activated Protein (MAP) Kinases, particularly Jun Kinase (JNK)1, and the induction of NLRP3 inflammasomes (Figure 1C) [4,20,21]. This is of special interest, since patients with sporadic CNO/CRMO also present anomalies in the MAP kinase pathways of monocytes (see below). Lipopolysaccharide (LPS) treatment of human or murine monocytes that either expressed increased or reduced amounts of Lipin2 produced altered levels of TNF-α, IL-6 and the chemokine C-C motif ligand (Ccl-)2. Cells with increased Lipin2 expression failed to produce pro-inflammatory cytokines, whereas reduced Lipin2 expression mediated increased expression of TNF-α, IL-6, and Ccl-2. Thus, Lipin2 may be a negative regulator of fatty acid induced pro-inflammatory signaling in monocytes and macrophages [2-4].

Patients with Majeed syndrome moderately respond to treatment with NSAIDs and corticosteroids. The dyserythropoietic anemia, however, was not improved by treatment [2-4]. Recently, IL-1 blockade with anakinra (IL-1RA) and canakinumab (an IL-1β antibody) has been demonstrated efficacious. Treatment benefits support the aforementioned hypothesis of LPIN2 mutations being associated with increased inflammasome activity and IL-1 release. Even though anemia improved with IL-1 blockade, it remains unclear whether dyserythropoiesis resolved since bone marrow biopsies have not been performed after treatment initiation [22].

## Sporadic chronic recurrent multifocal osteomyelitis (CRMO)

Chronic nonbacterial osteomyelitis (CNO) is a non-infectious inflammatory disorder of yet to be determined etiopathology. It represents a clinical spectrum with self-limited mono- or oligo-focal inflammatory bone involvement on one end and chronic recurrent courses on the other end of the spectrum, which are then referred to as CRMO [2-4].

Children and adolescents are most commonly affected. Symptoms at disease onset can be mild with bone pain with or without local swelling and warmth, or acute with more severe pain, malaise, (usually mild) fevers and even fractures. While, as aforementioned, in some patients inflammation can be self-limited and mono- or oligo-focal, a subset of patients develop chronic disease with ongoing inflammation over years and may suffer from sequelae, such as vertebral fractures and gibbus formation. Bone lesions can occur at any site of the skeleton except for the neurocranium. Additional organs can be involved, including the skin, the eyes, the gastrointestinal system, and the lungs. Skin inflammation can manifest as palmoplantar pustulosis, psoriasis, and occasionally as pyoderma gangrenosum [3,4]. This is of special interest, since most of the skin manifestations of CNO/CRMO also occur in the aforementioned monogenic autoinflammatory disorders. Furthermore, CNO can be part of the so-called SAPHO syndrome (Synovitis, acne, pustulosis, hyperostosis, and osteitis), which manifests in adolescence or adulthood. Whether CRMO in juvenile patients and SAPHO syndrome are the same disorder in different age groups remains to be determined [2-4]. Up to 60% of (usually adolescent or adult) patients may resemble or evolve into spondylarthropathies [23].

The precise pathophysiology of CNO/CRMO remains unknown. However, recent findings from our group and others indicate that an imbalance between pro- (IL-6, TNF-α) and anti-inflammatory (IL-10) cytokines may be centrally involved in the molecular pathology of CNO. In the serum of newly diagnosed and untreated CRMO patients, levels of IL-6 and TNF-α were elevated while IL-10 was not detectable in a single patient included in our study [24]. Furthermore, monocytes from CRMO patients failed to produce IL-10 under resting conditions and after stimulation of TLR4 with lipopolysaccharide (LPS) [24]. Since IL10 is regulated on the transcriptional level by promoter haplotypes influencing recruitment capacities of the transcription factor specificity protein (Sp-)1 to its binding elements [25], we asked whether such promoter variants may be responsible for the failure to produce IL-10 by CRMO monocytes. Interestingly, in CRMO patients such IL10 promoter haplotypes that encode for “high” (GCC) rather than “low” (ATA) IL-10 expression were enriched [24,26]. The failure to express IL-10 was linked with reduced activation of the MAP kinases Extracellular Signal Regulated kinase (ERK)1 and ERK2 which in turn results in reduced recruitment of the transcription factor Sp-1 to the IL10 proximal promoter and impaired histone H3 serin 10 phosphorylation (H3S10P), an activating epigenetic mark that had previously been demonstrated to be central for IL-10 expression in monocytes and macrophages (Figure 1D) [24,27]. Activating epigenetic patterns allow for transcription factor recruitment and the initiation of transcriptional complex formation by the rearrangement of nucleosomes. However, at this point, it remains to be determined which exact molecular defect results in the failure to activate ERK1/2 in response to TLR4 stimulation. Interestingly, the expression of pro-inflammatory cytokines IL-6 and TNF-α is not affected by this molecular defect, which is most likely caused by the fact that the induction of the p38 MAPK and JNK was not affected, resulting in an imbalanced activation of p38 and JNK on the one and ERK1/2 on the other hand [24,27]. This is of particular interest, since imbalanced MAPK induction can also be caused by an increased activation of JNK in response to TLR2/4 activation. This mechanism subsequently contributes to increased NFkB recruitment and pro-inflammatory cytokine expression. Therefore, this mechanism may be of central relevance for the molecular pathology of inherited familial osteomyelitis, since decreased expression of LPIN2, which is the cause of Majeed syndrome, results in increased expression of proinflammatory cytokines including TNF-α and IL-6. Conversely, forced expression of LPIN2 resuls in reduced inflammatory responses [4]. In this context it may also be of interest that infections with Propionibacterium acnes or other bacterial pathogens (Bartonella) have been suspected to trigger CNO/CRMO [28,29]. Next to TLR9, P. acnes may also activate TLR2 and TLR4 signaling, resulting in MAP kinase activation. Thus, we hypothesize that the imbalance between the expression of IL-10 and pro-inflammatory cytokines may be central to the pathophysiology of CRMO [30,31]. TNF-α and IL-6 contribute to bone resorption and remodeling by inducing the receptor activator of NFkB (RANK) ligand on stromal cells favoring osteoclast differentiation and activation, while IL-10 can suppress RANK-induced effects on osteoclasts [3,30,32-36].

Familial clusters with affected siblings and parents together with an increased prevalence of other inflammatory diseases, such as inflammatory bowel disease, in patients and first-degree relatives, suggest a genetic component to CNO/CRMO. Still, to date no single gene variant has been linked to the onset of sporadic CRMO [3-5]. A CRMO susceptibility locus has been mapped to chromosome 18q21.3-22 [37] and the aforementioned enrichment of “GCC” IL10 promoter haplotypes that encode for “high” IL-10 expression further suggests a genomic component to CNO [24,26]. However, both findings remain to be established in larger cohorts from other backgrounds. Polymorphisms in genes that co-segregate with related inflammatory conditions were not present in CNO/CRMO (PSTPIP1, PSTPIP2, LPIN2, IL1RN, CARD15), indicating that CRMO and those inflammatory disorders may be related and share key symptoms but are still distinct from one another [38-40]. Identifying common pathways and differences in pro-inflammatory signaling may prove helpful in the search for molecular mechanisms of cytokine deregulation in CNO/CRMO.

Sporadic CNO/CRMO is a diagnosis of exclusion, since the clinical presentation and laboratory findings are not disease-specific and vary between affected individuals. Differential diagnoses include infections (mycobacteria, septic osteomyelitis, etc.), malignancies (bone tumors, metastases, leukemia or lymphoma), benign tumors (osteoid osteoma, bone cysts, fibrosis), further autoinflammatory disorders (PAPA, DIRA, Majeed syndrome, etc.), osteonecrosis, osteopetrosis, and others. Laboratory findings include (slightly) elevated inflammation markers (CrP, ESR, IL-6, TNF-α) in the absence of high-titer auto-antibodies. A subset of patients, however, do not exhibit any serological prove of systemic inflammation. Genomic markers are not available for the diagnosis of CNO/CRMO, since no HLA-associations or single gene mutations have been reported. At this point, no specific biomarkers for the diagnosis and/or measurement of disease activity are available [2-4].

Inflammatory bone lesions may be detected in plain radiographs presenting as radiolucent, osteolytic or sclerotic lesions, depending on the disease stage. However, in early phases of CNO/CRMO radiographs may remain normal. Whole-body imaging techniques, including MRI and Tc-99 m labeled methylene bone scintigraphy, have become widely available and represent novel and valuable diagnostic tools. Since CNO/CRMO reconstitutes a systemic disease with potentially multiple inflammatory lesions, whole-body imaging techniques should be performed at least at the time of diagnosis to exclude asymptomatic lesions e.g. in the vertebral column. MRI is especially sensitive during early stages, since it can detect bone edema even before erosions and/or sclerosis become apparent. Furthermore, MRI techniques allow an assessment of adjacent tissues. At the time of diagnosis, gadolinium-enhanced T1 or strongly T2 weighted sequences (Turbo Inversion Recovery Measurement; TIRM) with fat saturation can be used and are superior to conventional radiographs or scintigraphy. However, diagnoses can be complicated by low specificity of radiological findings, especially during phases of clinical remission in children. For follow-up non-enhanced sequences may be sufficient [41,42].

**Figure 2:**
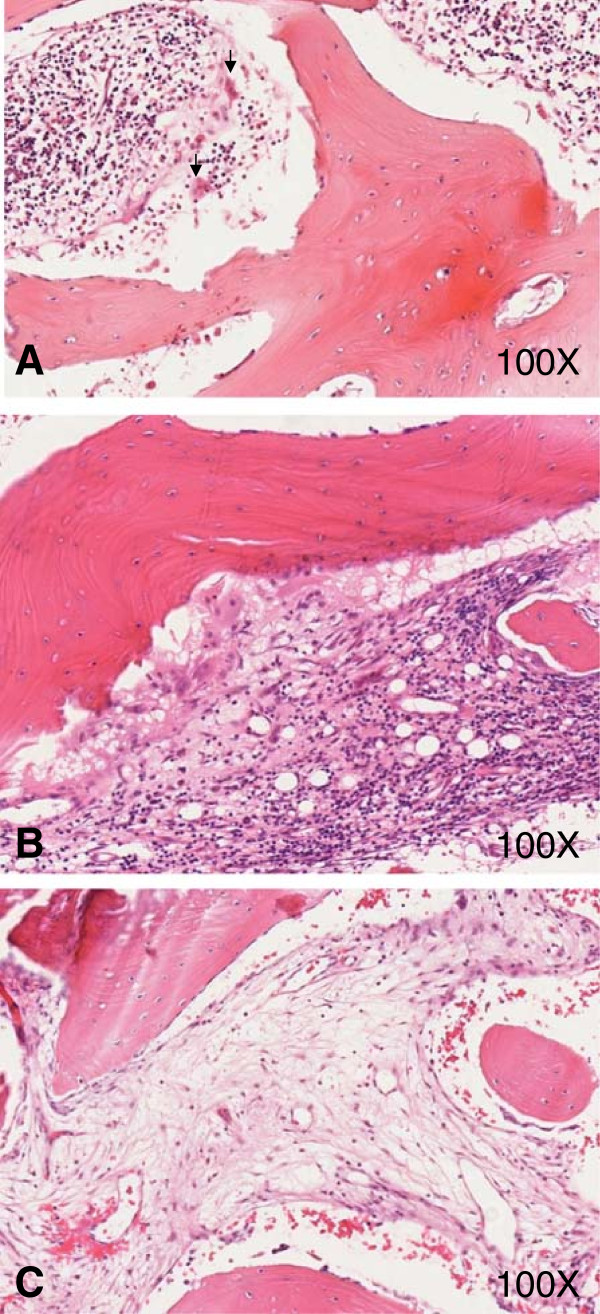
**Histological appearance of early and late stages of CRMO. A)** Hematoxylin and eosin (HE) stained bone biopsy in the early phase of CRMO. Neutrophils and monocytes (arrows) are the predominant cell types. **B)** HE stained bone biopsy showing chronic inflammation in CRMO with monocytes, lymphocytes and plasma cell infiltration. **C)** HE stained biopsy of the late chronic fibrosing stage in CRMO.

In unclear cases; especially such with monofocal bone lesions in untypical sites, fevers, highly elevated inflammatory markers (CrP, ESR) and/or pathological white blood cell differentiation patterns, or elevated levels of uric acid or LDH; bone biopsies should be performed to exclude the aforementioned differential diagnoses. In CNO/CRMO, cellular infiltrates are highly dependent on the disease stage. In early phases, neutrophils are the predominant cell subset (Figure 2A). Monocytes, macrophages, lymphocytes, and plasma cells can be detected during later stages of the disease (Figure 2B). Osteolyses and concomitant slcerosis and/or fibrosis can be seen in late phases of CNO/CRMO (Figure 2C). The cellular distribution in inflammatory lesions reflects the immunological reaction at the time. Innate immune activation in early disease stages may be reflected by neutrophils, macrophages and monocytes, while subsequent activation of the adaptive immune system may be reflected by the presence and later predominance of lymphocytes and plasma cells [43]. Unfortunately, very little is known about the kinetics of inflammatory responses. Based on our preliminary data from biopsies collected from 12 CRMO patients [43], we conclude that early inflammation may last for approximately six months, followed by the lymphocytic/plasma cellular phase that may last for several months or years. The fibrosing end-stage may be reached after several years of active bone inflammation [43].

**Figure 3:**
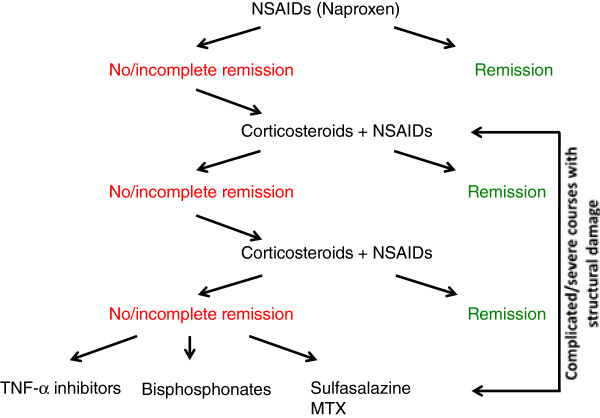
**Treatment of CNO/CRMO.** NSAIDs, preferentially naproxen, should be applied as first-line therapy for most patients. When disease activity is high or complications, such as vertebral involvement or fractures, are present at the time of diagnosis, corticosteroids or biological treatment may be considered. Treatment effects should be monitored after three months, using MRI. If patients fail to respond within three months, treatment can be escalated. The authors apply 2 mg/kg oral prednisone per day over two weeks, followed by clinical assessment and MRI imaging after three months. Our treatment goal is clinical, and, in the case of vertebral involvement, complete radiological remission. In the case of a relapse after initial improvement, we repeat high-dose steroids (2 mg/kg/day) for seven days or apply low-dose corticosteroids (0.1-0.2 mg/kg/day) over a longer period. In individuals who relapse again or fail to reach clinical and (if vertebrae are involved) radiological remission, TNF-α inhibitors or bisphosphonates should be considered. Though, reports on the use of sulfasalazine and particularly methotrexate are limited, we made favorable experience using sulfasalazine in the treatment of concomitant inflammatory bowel disease, skin involvement, or HLA-B27 positivity.

Treatment in CNO/CRMO is empiric, since placebo controlled randomized trials have not been performed [3,4,39,43]. NSAID are commonly used as first-line treatment of CNO/CRMO in less to moderately severe cases. NSAIDs provide pain control in most cases and most likely alter the disease course, since prostaglandins are centrally involved in osteoclast activation and bone remodeling. Recently, we reported a cohort of CNO/CRMO patients treated with naproxen over one year. In over half of our patients, a symptom free status was achieved after 12 months. However, complete clinical remission in the absence of radiographic proof of inflammation was only achieved in 27% of patients [44]. In a recent study, response rates to NSAIDs were reported lower (13%) [45]. Differences between this US American and our study may be due to the collection of retrospective data from charts in two independent sites over a 24-year interval, and the incomplete availability of results from imaging techniques. For the same reasons, patients with more severe and treatment-resistant forms of CNO/CRMO may be over-represented in this cohort. In our prospective study, predictors of incomplete response to NSAIDs were arthritis and/or vertebral involvement at diagnosis [44]. Over 40% of our patients developed radiographic signs of recurring bone inflammation after the discontinuation of naproxen, 67% of which remained clinically silent [44]. Over all, our data suggest a favorable response to NSAIDs in most patients. The discrepancy between clinical and radiographic remission raises the questions of when NSAID treatment can be discontinued safely and whether treatment is necessary in the absence of clinical symptoms. Both questions are of special relevance, since it appears likely that CNO/CRMO is largely under-diagnosed. This may be secondary to the absence of subjective complains in a subset of patients, which only get diagnosed after vertebral fractures that may be prevented by early treatment. In our study and according to other reports, a significant fraction of CNO/CRMO patients require more aggressive treatment regimens [4,44,46]. Second line options include corticosteroids, sulfasalazine, methotrexate (MTX), anti-TNF agents (usually etanercept or infliximab), or bisphosphonates (usually pamidronate) [3,4]. Case reports and our clinical experience suggest favorable effects of corticosteroids in a majority of patients (60%) reviewed in [3,5]. Unfortunately, prospective studies are not available and safety concerns need to be considered [3,4]. Thus, we recently proposed a therapeutic escalation regimen based on (the limited) published data on treatment response in CNO/CRMO and our own experience (Figure 3) [47]. In NSAID refractory cases, authors apply corticosteroids in high-dose regimens (up to 2 mg/kg/d) for two weeks or continuously in lower doses (0.1-0.2 mg/kg/day). Sulfasalazine has been reported beneficial in case reports and case series. Recently, Borzutzky et al. reported limited effects in most patients (clinical remission in 18%) [45]. However, the authors’ personal experience suggests higher efficacy in selected CNO/CRMO subsets, particularly HLA-B27 positive patients and in patients with concomitant inflammatory bowel disease [3,4,48]. MTX is a relatively common treatment choice for CNO/CRMO in North America. However, two recent reports suggest the induction of clinical remission in only a subset of patients (up to 20%) [45,49]. Given the imbalance between pro- (IL-6 and TNF-α) and anti-inflammatory cytokines in CNO/CRMO, TNF-α blockers have proven useful in a subset of patients (up to 65%) [4,44,46]. However, a significant fraction of treated individuals do not fully respond. Though most CNO/CRMO patients receiving anti-TNF treatment were resistant to standard treatment, more than one third did also not completely respond to TNF inhibitors. Given the off-label character and the high cost of TNF-α blocking agents, together with safety concerns, they should be reserved for complicated cases with structural damage and/or steroid refractory courses [3,44,46]. Intravenous application of bisphosphonates has proven effective in pain reduction and bone-inflammation (up to 80% have been reported to experience improvement) and some patients reach complete remission after a single dose reviewed in [3,5,50-53]. However, most individuals require repeated infusions to reach and sustain remission. Usually three to four courses are applied in four-weeks intervals reviewed in [3,5,50-53]. Given potential safety concerns during long-term treatment in children and adolescents, bisphosphonates should be reserved for severe cases (with structural damage, e.g. vertebral fractures) or in otherwise treatment-resistant patients.

## CNO/CRMO in animals

Genetically determined CNO has been reported in animals which function as disease models, helping to understand molecular requirements and immunological patterns of bone inflammation.

Severe CRMO with systemic inflammation occurs in mice with mutations in the proline serine threonine interacting protein 2 (Pstpip2) gene. Two strains have been reported: Chronic multifocal osteomyelitis (CMO) mice carry a spontaneous mutation in Pstpip2 (p.L98P) [54], in lupo mice a Pstpip2 mutation was chemically induced (p.I282N) [55]. Both mice develop phenotypes that are similar to severe human CRMO with multiple sites of bone inflammation. Since both total (in CMO mice) and partial (in lupo mice) PSTPIP2 deficiency result in a pro-inflammatory phenotype, PSTPIP2 has been suggested to exert anti-inflammatory functions. Unlike most human CRMO patients, CMO and lupo mice exhibit additional signs of systemic inflammation, including hepatosplenomegaly, extramedullary hematopoiesis, and elevated serum inflammatory parameters (MIP-1a, RANKL, both osteoclastogenic factors, IL-1α, IL-1β, TNF-α, and IL-6) [54,55]. Cross breeding with RAG^−/−^ mice demonstrated disease expression independent from adaptive immune cells [55]. Macrophage depletion or treatment with inhibitors of the M-CSF receptor (c-FMS), decreased circulating MIP-1a and diminished extramedullary hematopoiesis and inflammation. Furthermore, osteoclast precursor cells in CRMO-prone mice exhibit increased osteoclastogenesis in vitro. This suggests that cytokine imbalance in CRMO-prone mice drives osteoclast generation and activation, resulting in sterile inflammation and bone loss [54]. Taken together, over-responsive myeloid cells and subsequent osteoclast generation and activation have been suggested to be major contributors to the pro-inflammatory phenotype of CMO and lupo mice. This is of special interest, since this mechanism may also be central to the molecular pathogenesis in human CRMO.

Another form of CRMO in animals is referred to as hypertrophic osteodystrophy (HOD), which is prevalent in large breed dogs. HOD usually presents in young dogs with fevers and bone pain. Similar to human CRMO, vertebral bodies and the metaphyses of long bones are most commonly affected. Osteolytic and sclerotic lesions can be detected in radiographs. In biopsies sterile osteomyelitis and osteonecrosis can be seen. Extra osseous manifestations include skin rashes and diarrhea [56,57]. Even though self-limiting in most cases, sometimes HOD can take a life-threatening course. Affected animals respond to NSAIDs but corticosteroids show better efficacy. The etiopathology of HOD remains unclear. Since specific large breeds are most commonly affected and familial cluster have been reported, an autosomal-recessive inheritance or dominant traits with incomplete penetrance are most likely. One study in affected Weimaraner dogs documented an association of HOD with DLA-DRB1*01501 that was not reproduced in follow-up investigations [56,57].

## Conclusions

Over the past decade, significant progress has been made in understanding the pathophysiology of autoinflammatory bone disorders in humans and animals. Regardless of the identification of disease-causing genes in some monogenic disorders, the exact pathophysiology remains largely elusive for most of them (with the exception of DIRA). In the light of in vivo and in vitro studies, together with favorable response to cytokine blocking strategies, an imbalance between pro- and anti-inflammatory cytokines appears to be central for disease expression in all of them.

In the (most probably) polygenic human disorder CNO/CRMO, progress has been made targeting molecular pathomechanisms and molecular disturbances. Defective MAP kinase pathways result in impaired anti-inflammatory cytokine expression. Disease-causing mutations in one or multiple genes, however, remain to be determined. The clinical picture and outcomes in CRMO are highly variable, complicating the identification of patients who require treatment on top of NSAIDs to control disease activity and prevent complications. Thus, the development of clinical scoring systems and biomarkers will prove essential in order to select patients at risk for fractures and/or prolonged disease courses.

## Competing interests

The authors declare that they have no competing interests.

## Authors’ contribution

All authors contributed to the drafting of the manuscript and approved the final version. JP provided histologies from bone biopsies of CNO patients.
